# Heterogeneous Nitrogen Supply With High Frequency and Ramet Damage Increases the Benefits of Clonal Integration in Invasive *Hydrocotyle vulgaris*

**DOI:** 10.3389/fpls.2022.825492

**Published:** 2022-04-29

**Authors:** Kai Sun, Jing-Fang Cai, Yu Zhang, Ya-Nan Mu, Si-Ha A, Yi-Luan Shen, Li-Juan Yang, Hong-Li Li

**Affiliations:** School of Ecology and Nature Conservation, Beijing Forestry University, Beijing, China

**Keywords:** herbivory, invasive plant, physiology integration, stable isotope, wetland plant

## Abstract

Nitrogen (N) deposition significantly affects the growth and the function of invasive clonal plants. However, the effects of heterogeneous N supply with different frequencies on the growth and the potential contribution of clonal integration in invasion plants are still unclear, especially in the complex environment considering ramet damage. To address this question, apical and basal ramets of the clonal invader *Hydrocotyle vulgaris* were connected or disconnected, N was added to the basal ramets with a high frequency, a low frequency, or no supply, and the total N quantity was the same for the different frequency. Furthermore, 8 aphids were placed on the apical ramets, and 30% of each leaf was cut off to cause damage. The connection between ramets significantly increased the biomass, total carbon (C), and total N of the basal and apical ramets. Higher frequency N supply significantly increased the biomass, total C, and total N of the basal ramets and the entire clonal fragment biomass. The damage had no significant effect on the growth of basal and apical ramets. Especially, under the high N frequency and ramet damage condition, the connection between ramets more significantly increased the biomass, total C, and total N of the apical ramets and the entire clonal fragment biomass. In addition, the uptake rates of ^15^${\text{NH}}_{4}^{+}$ and ^15^${\text{NO}}_{3}^{-}$ in *H. vulgaris* had no significant difference, and N supply increased the uptake rates of ^15^${\text{NH}}_{4}^{+}$ and ^15^${\text{NO}}_{3}^{-}$ of the basal ramets. Our results suggest that both higher frequency N supply and clonal integration are beneficial to the growth of *H. vulgaris*. Moreover, the heterogeneous N supply with high frequency and ramet damage increases the benefits of clonal integration in *H. vulgaris*. These findings improve our understanding of the response of clonal invader *H. vulgaris* to nitrogen deposition and ramet damage.

## Introduction

Alien plants can quickly adapt to new environments, replacing the local plants and seriously damaging the local ecosystem due to some specific traits (Richardson et al., 2000; Kleunen et al., 2010). Studies have shown that clonal integration may be an important trait for alien plants to quickly adapt and successfully invade new environments (Liu et al., 2006; Yu et al., 2009; Song et al., 2013). Besides, due to the influence of fertilization, disturbance, and soil properties, the distribution of soil nutrients needed for plant growth is often heterogeneous in habitats (Zhang et al., 2016; Shen et al., 2019). In clonal plants, heterogeneous resources and colonization of habitats are moderated through clonal integration, where water, nutrients, and carbohydrates are translocated among ramets through a connecting rhizome or stolon, subsequently promoting their growth (Wei et al., 2019; Yu et al., 2019; Zhang et al., 2019; Franklin et al., 2020).

Due to human activities, the total amount of atmospheric nitrogen (N) deposition and the rate keep increasing, which significantly affects the growth and function of plants (Gutiérrez, 2012; Peñuelas et al., 2012; Valliere and Allen, 2016). Previous studies showed that N addition can improve the division of labor of invasive clonal plants and promote their growth (Huang et al., 2018; Lin et al., 2018). In addition, it has been reported that the clonal integration benefits of clonal plants in heterogeneous N environments are more significant (Dong et al., 2015; Liu et al., 2017a; Ying et al., 2018). However, previous studies on the N environment and clonal plants were only based on the supply level of N (Huang et al., 2018; Lin et al., 2018; Dong et al., 2019). In fact, N deposition in these environments is a continuous process, and the frequency of deposition also significantly impacts plant growth (Carreiro et al., 2000; Phoenix et al., 2004). A single high amount of N addition amplifies the ecosystem pulse effect in the short term and weakens the long-term impact of N deposition (Moldan et al., 2018). Thus, we should consider the potential effects of N supply frequency when exploring the responses of plant growth to simulated N deposition (Cao et al., 2020, 2021). This study aims to provide an experimental test for the effects of heterogeneous N supply with different frequencies on the growth and the clonal integration of clonal plants.

Besides heterogeneous N resources, studies have shown that ramet damage also significantly affects the growth and the clonal integration of cloned plants (Hellström et al., 2006; Liu et al., 2007). For clonal plants, under the action of clonal integration, plants can deal with the ramet damage through overall resource allocation (Liu et al., 2009; Tewari et al., 2014). For example, damage can also be transmitted between ramets as a signal so that normal ramets can deal with damage in advance (Hettenhausen et al., 2017; Zhuang et al., 2018). In addition, a recent study shows that the damage of ramets may also induce the negative effects of clonal integration (Gao et al., 2021). However, less is known about the interaction effects between N deposition and ramet damage on the growth of cloned plants and their clonal integration (Dong et al., 2019).

In addition, the translocation of resources among ramets is not equal, so clonal integration has different effects on different ramets (Salzman and Parker, 1985; Gao et al., 2014; Wang et al., 2017). Generally, all ramets will benefit from clonal integration due to the rational division of labor and resource integration among ramets (Roiloa and Retuerto, 2007; Zhang et al., 2009). However, under some conditions, low resource ramets do not always obtain support from high resource ramets (Klimeš and Klimešová, 1999; Hay and Kelly, 2008). Besides, the reallocation of resources from the high- to the low-resource or damaged ramets may also result in neutral or negative effects on the high resource ramets (Yu et al., 2002; Pauliukonis and Gough, 2004; Wang et al., 2009; Chen et al., 2015). Therefore, it is necessary to quantify the effects of heterogeneous N supply and damage on ramets located in different environments.

To explore the response mechanism of the growth and the clonal integration of invasive clonal plants under heterogeneous N supply with different frequencies and ramet damage conditions, the clonal invader *Hydrocotyle vulgaris* was used as the model plant in a control experiment. Apical and basal ramets of *H. vulgaris* were connected or disconnected, N at different frequencies was added to the basal ramets, 8 aphids were placed on the apical ramets, and 30% of each leaf was cutoff to cause damage. We measured the morphological and physiological indexes of *H. vulgaris*, including the isotopic identification of the ^15^N-${\text{NH}}_{4}^{+}$ and ^15^N-${\text{NO}}_{3}^{-}$. Specifically, we addressed the following two questions: (1) How do the clonal integration and heterogeneous N supply with different frequencies and ramet damage affect the growth of *H. vulgaris*? (2) How do the heterogeneous N supply with different frequencies and ramet damage affect the benefits of clonal integration in *H. vulgaris*?

## Materials and Methods

### Experimental Materials

*Hydrocotyle vulgaris* L. is a native perennial herb in the family Apiaceae, growing in moist to wet habitats across Europe and northwestern Africa (Dong et al., 2015; Wang et al., 2018, 2020). It was introduced to China as a garden plant in artificial wetlands in the 1990s, from which it spread into natural habitats (Liu et al., 2014). *H. vulgaris* is a typical clonal plant with high phenotypic plasticity, strong clonal ability, and wide tolerance to habits. It can occupy a wider ecological amplitude than the native species (Wang et al., 2018, 2020). In our study, *H. vulgaris* was collected from the Xixi wetlands in Hangzhou, Zhejiang Province, China, in May 2015. The collected samples were vegetatively propagated in a greenhouse at the Forest Science Company, Ltd. of Beijing Forest University, Beijing, China.

The green peach aphid, *Myzus persicae* Sulzer, is a small, euryphagic, piercing, and sucking insect of the family Aphididae that infests the majority of agricultural crops and wild plants globally (Goggin, 2007). Its piercing-sucking phenomenon contributes to the rapid spread of plant viruses, such as *Potato virus* and *Turnip yellows virus* (Mondal and Gray, 2017; Congdon et al., 2019), which significantly affect the productivity of agricultural and forestry crops (Guerrieri and Digilio, 2008). A recent study showed that aphids can also cause some damage to *H. vulgaris* (Liu et al., 2017a). In our study, aphids were collected from *Rosa chinensis* Jacq in the greenhouse at the Forest Science Company, Ltd. of the Beijing Forest University in May 2015. They were then multiplied using *H. vulgaris* as the host plant for 1 year. Aphids from a single *H. vulgaris* clonal fragment were selected in this study.

### Experimental Design

**Figure 1:**
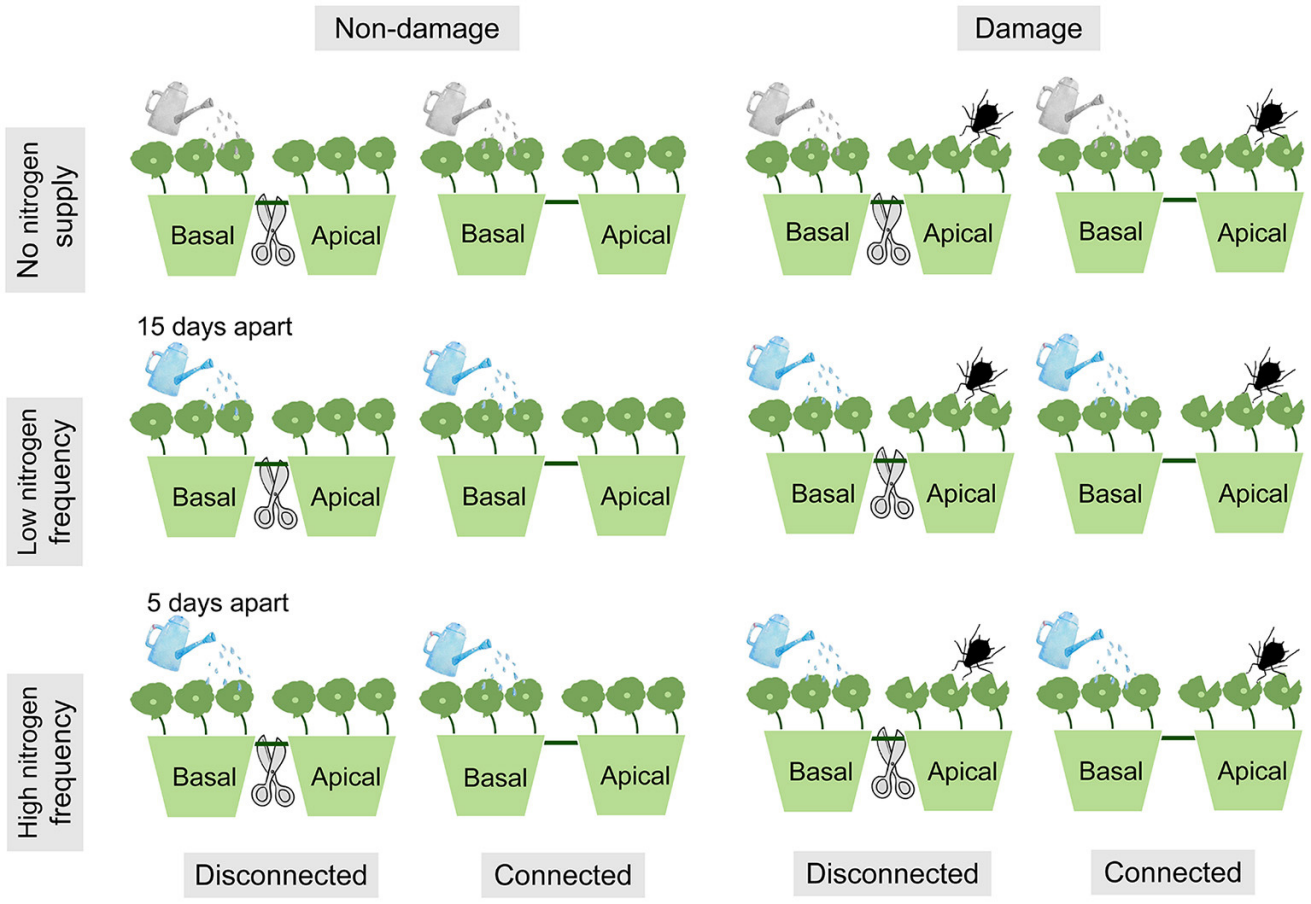
Experimental design. The ramets in each pot were connected. All treatments were sprayed with 100 ml of deionized water every 5 days. In addition, 0.039 g NH_4_NO_3_ was added to deionized water every 15 days at low nitrogen (N) frequency, and 0.013 g NH_4_NO_3_ was added to deionized water every 5 days at high N frequency.

The experiment used a fully factorial design consisting of three N frequency treatments crossed with two damage treatments (ramets damage or non-damage) and two connection treatments (clonal fragment connected or disconnected) (Figure 1). Each *H. vulgaris* fragment has 6 nodes, and every 3 nodes were planted in pots that had 17 cm inner diameter, 19 cm outer diameter, and 13 cm height. The older and younger ramets represented the basal and apical ramets, respectively. Since aphids prefer to feed on young ramets, we addeded aphids to the apical ramets (Gao et al., 2021). Corresponding to the damage, N was added to the basal ramets. In addition, connection or disconnected treatments were carried out in the middle parts of the fragments.

The three N supply frequencies were high frequency, low frequency, and no N supply on the basal ramets. All treatments were sprayed with 100 ml of deionized water every 5 days. In addition, 0.013 g NH_4_NO_3_ was added to deionized water every 5 days at high N frequency, and 0.039 g NH_4_NO_3_ was added to deionized water every 15 days at low N frequency. The total N deposition for both the low N frequency and the high N frequency treatments was 15 g N m^−2^ a^−1^. The total amount and frequency of N in the experiment were set according to the atmospheric N wet deposition and the precipitation in the natural distribution area of *H. vulgaris* in China (Li et al., 2011; Zhou et al., 2015).

Ramet damage was carried out by aphids throwing and cutting leaves on the apical ramets. During the experiment, 8 aphids were released on the apical ramets and checked regularly to keep the number stable (Liu et al., 2017a). Meanwhile, the container where apical ramets are growing was covered with gauze cages (length, 25 cm; width, 25 cm; height, 50 cm) to prevent the spread of aphids between containers. In addition, considering that the purpose of our study was to test the response of *H. vulgaris* growth and clonal integration to N deposition and ramet damage, we are not concerned about the effects of aphids on *H. vulgaris*. Therefore, we also simulated the leaf damage of the animal and mechanical injuries to stimulate the *H. vulgaris* response to ramet damage. More specifically, we removed 30% of each leaf of all apical ramets on the 45th day after the start of the experiment (Portela et al., 2019).

We added ^15^NH_4_NO_3_ and ${\text{NH}}_{4}^{15}$NO_3_ isotopes 24 h before harvest. Six replicates were randomly and equally divided into two groups for the addition of ^15^NH_4_NO_3_ and ${\text{NH}}_{4}^{15}$NO_3_, respectively. To detect an appropriate amount of ^15^N in ramets after 24 h, we checked the ^15^N abundances in ^15^NH_4_NO_3_ and ${\text{NH}}_{4}^{15}$NO_3_, which were 99.11 and 99.23%, respectively, and the added total amount in each pot was 12.5 ^15^NH_4_NO_3_ or ${\text{NH}}_{4}^{15}$NO_3_ mg·m^−2^ (Gao et al., 2021). The applied isotope was dissolved into 100 ml distilled water and applied on the soil surface evenly using a needle tube.

The experiment started on 11 July 2016 in the same greenhouse where *H. vulgaris* was cultivated. There were 12 treatments in total, and there were 6 repetitions for each treatment. There were two pots in line together for each treatment to ensure that the basal and apical ramets are placed separately to facilitate the observation of clonal integration. The pots were filled with a 1:1:1 (v:v:v) mixture of peat:vermiculite:quartz sand, and some ceramsite were placed at the bottom of the pots to prevent soil loss. There were 144 pots in total for the experiment.

During the experiment, the mean temperature was 28.4 ± 0.3°C, and relative humidity was 64.4 ± 0.8%, as measured by I Buttons (DS1923; Maxim Integrated Products, Sunnyvale, CA, USA).

### Morphological Measurements

Plants of *H. vulgaris* were harvested on 15 October 2016. The clonal fragments in each combination pot were separated into two portions. The basal and apical ramet portions consisted of the original ramet and any new stems and ramets it had produced.

Within each portion of ramets, the numbers of leaves and nodes were counted, and the total stem length was measured. In addition, the total leaf area was measured using a Win FOLIA Pro 2004a (Regent Instruments, Inc., Canada). Plants were then divided into roots, stems, and leaves, dried at 75°C for 72 h, and weighed.

### Physiological Measurements

Recent studies have shown that N deposition and ramet damage not only affect plant morphological traits but also significantly affect plant physiological traits (Cao et al., 2021; Gao et al., 2021). Therefore, we measured the total carbon (C) and N of the basal and apical ramets to improve our understanding of the response of *H. vulgaris* to N deposition and ramet damage.

The total C and total N contents were determined with a total organic carbon (TOC) (multi N/C 3100, Analytik Jena, Germany) analyzer and a continuous flow analyzer (SEAL AA3, SEAL, Germany).

The ^15^N-${\text{NH}}_{4}^{+}$ and ^15^N-${\text{NO}}_{3}^{-}$ isotopes of apical and basal ramets were determined using the DELTAV Advantage Isotope Ratio Mass Spectrometer and the Flash 2000 HT Element Analyzer. Considering that there is no resource transfer between ramets when they are not connected (Gao et al., 2021), we did not measure the isotopes of apical ramets when they are not connected. The samples were burned at high temperatures in an elemental analyzer to generate N_2_. Then, the mass spectrometer calculated the δ^15^N values of the samples after detecting the ^15^N to ^14^N ratio of N_2_ and comparing it with the international standard (atmospheric N_2_). The determination accuracy was δ^15^N: ± <0.2‰.

### Statistical Analyses

**Figure 2:**
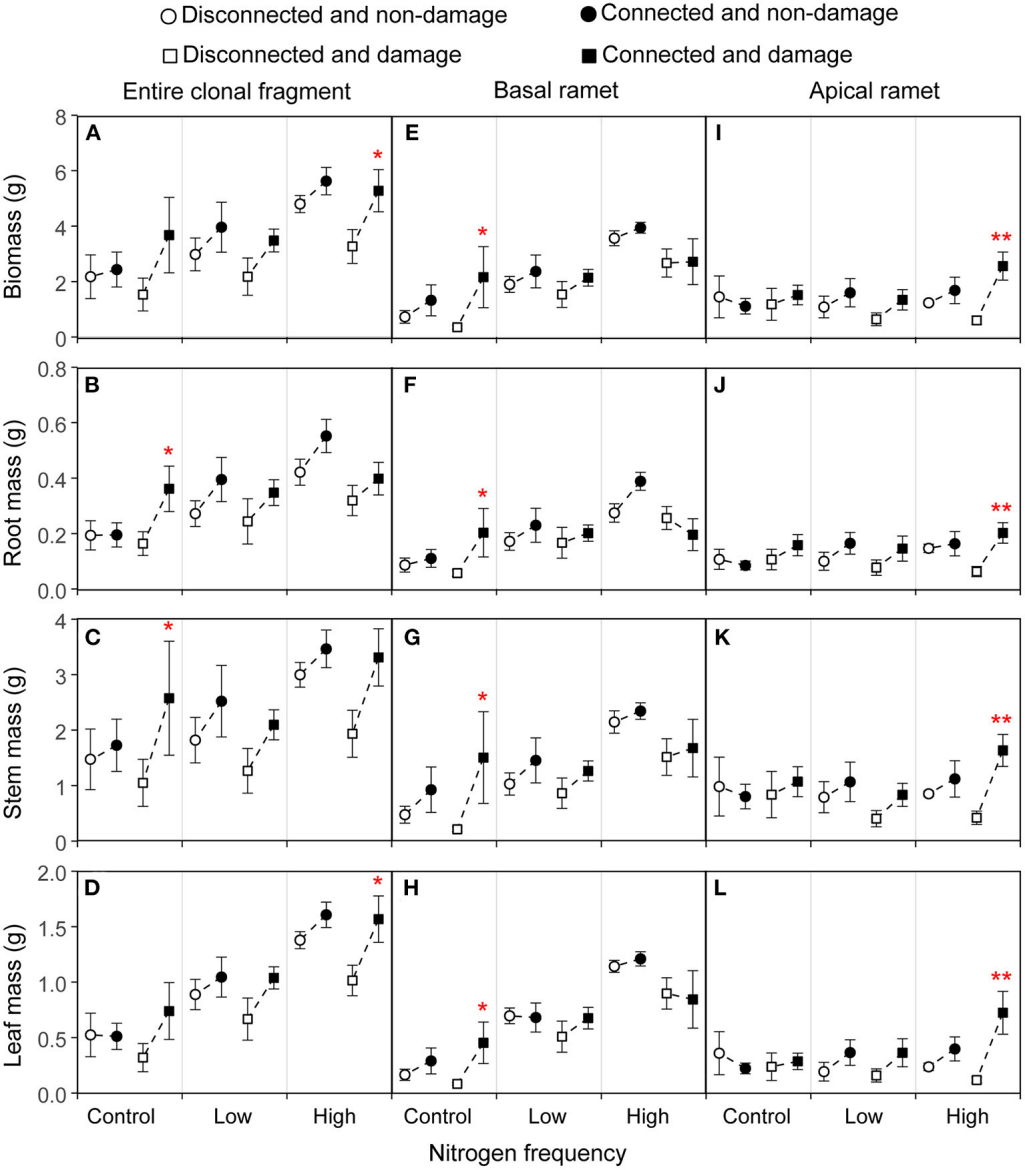
Effects of connection among ramets, N supply frequency, and damage on the biomass, root mass, stem mass, and leaf mass of the entire clonal fragment **(A–D)**, basal ramets **(E–H)**, and apical ramets **(I–L)** of *Hydrocotyle vulgaris*. Bars represent the mean ± SE. The *p*-values with significant difference between disconnected and connected treatments under each N frequency and damage combination (linear contrast based on ANOVA) were marked above the connected treatment. *P < 0.05; **P < 0.01. Refer to Table 1 for ANOVAs.

**Figure 3:**
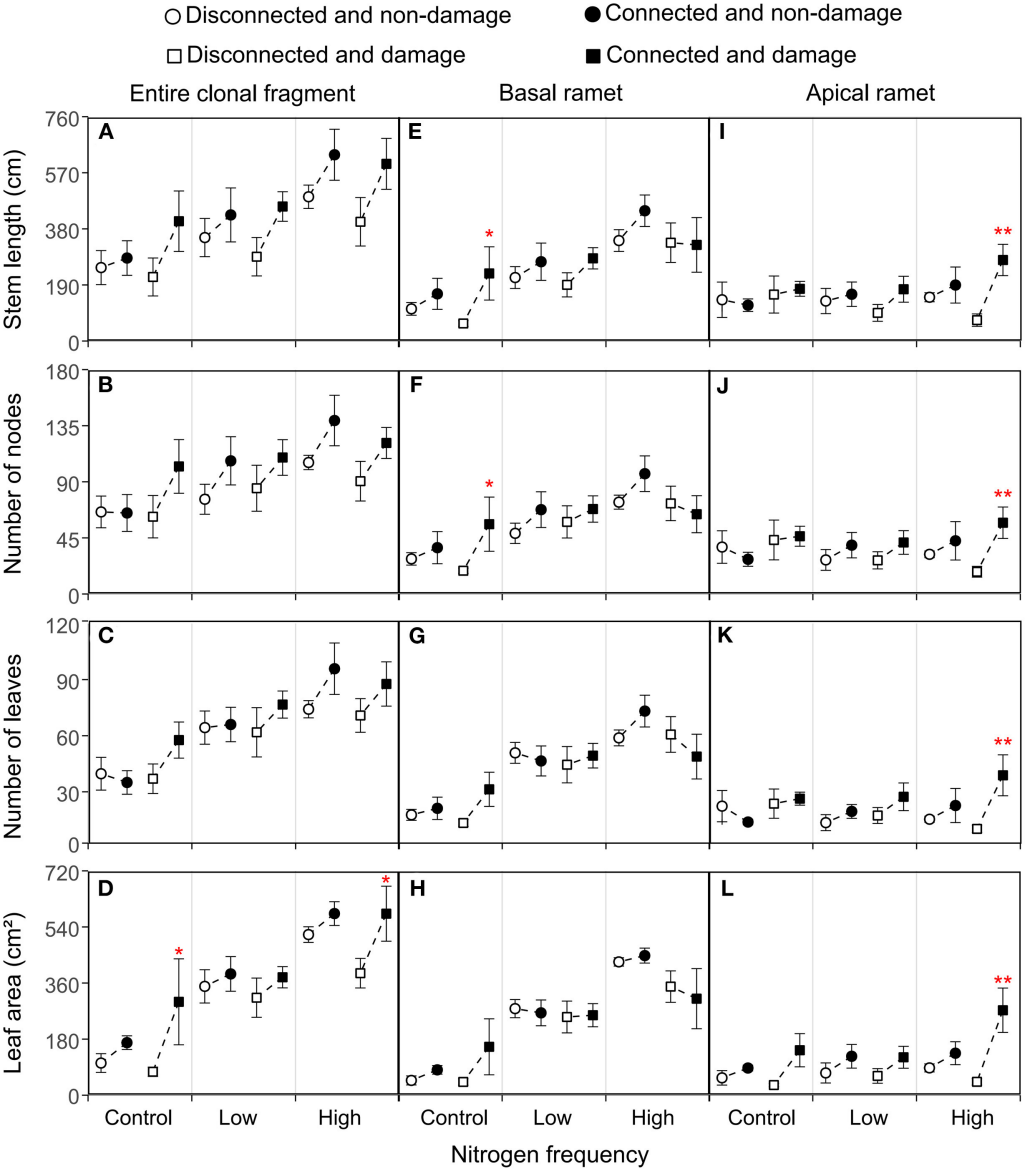
Effects of connection among ramets, N supply frequency, and damage on the stem length, number of nodes, number of leaves, and leaf area of the entire clonal fragment **(A–D)**, basal ramets **(E–H)**, and apical ramets **(I–L)** of *H. vulgaris*. Bars represent the mean ± SE. The *p*-values with significant differences between disconnected and connected treatments under each N frequency and damage combination (linear contrast based on ANOVA) were marked above the connected treatment. *P < 0.05; **P < 0.01. Refer to Table 2 for ANOVAs.

**Figure 4:**
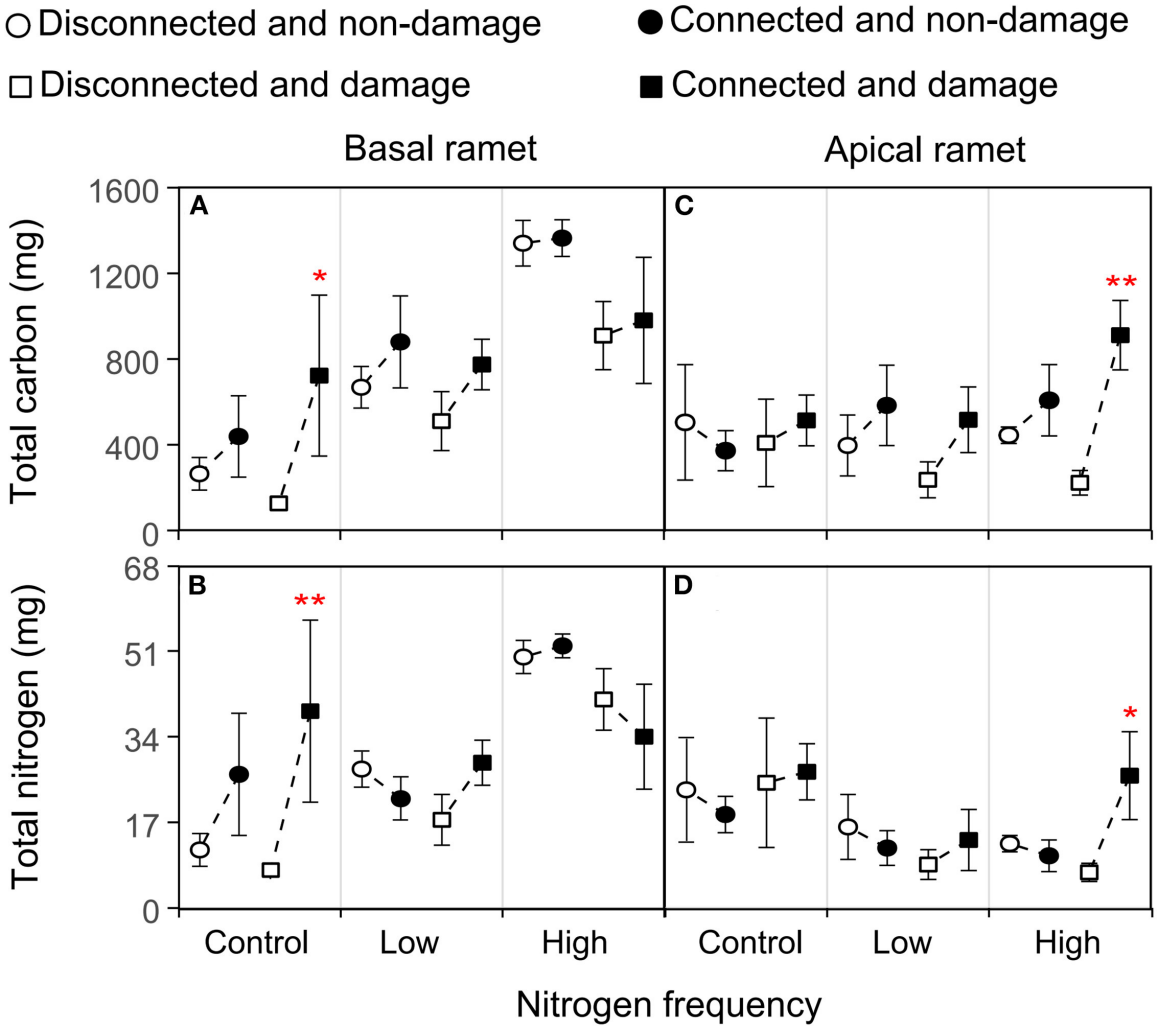
Effects of connection among ramets, N supply frequency, and damage on the total carbon and total N of the basal **(A,B)** and apical **(C,D)** ramets of *H. vulgaris*. Bars represent the mean ± SE. The *p*-values with significant differences between disconnected and connected under each N frequency and damage combination (linear contrast based on ANOVA) were marked above the connected treatment. *P < 0.05; **P < 0.01. Refer to Table 3 for ANOVAs.

**Figure 5:**
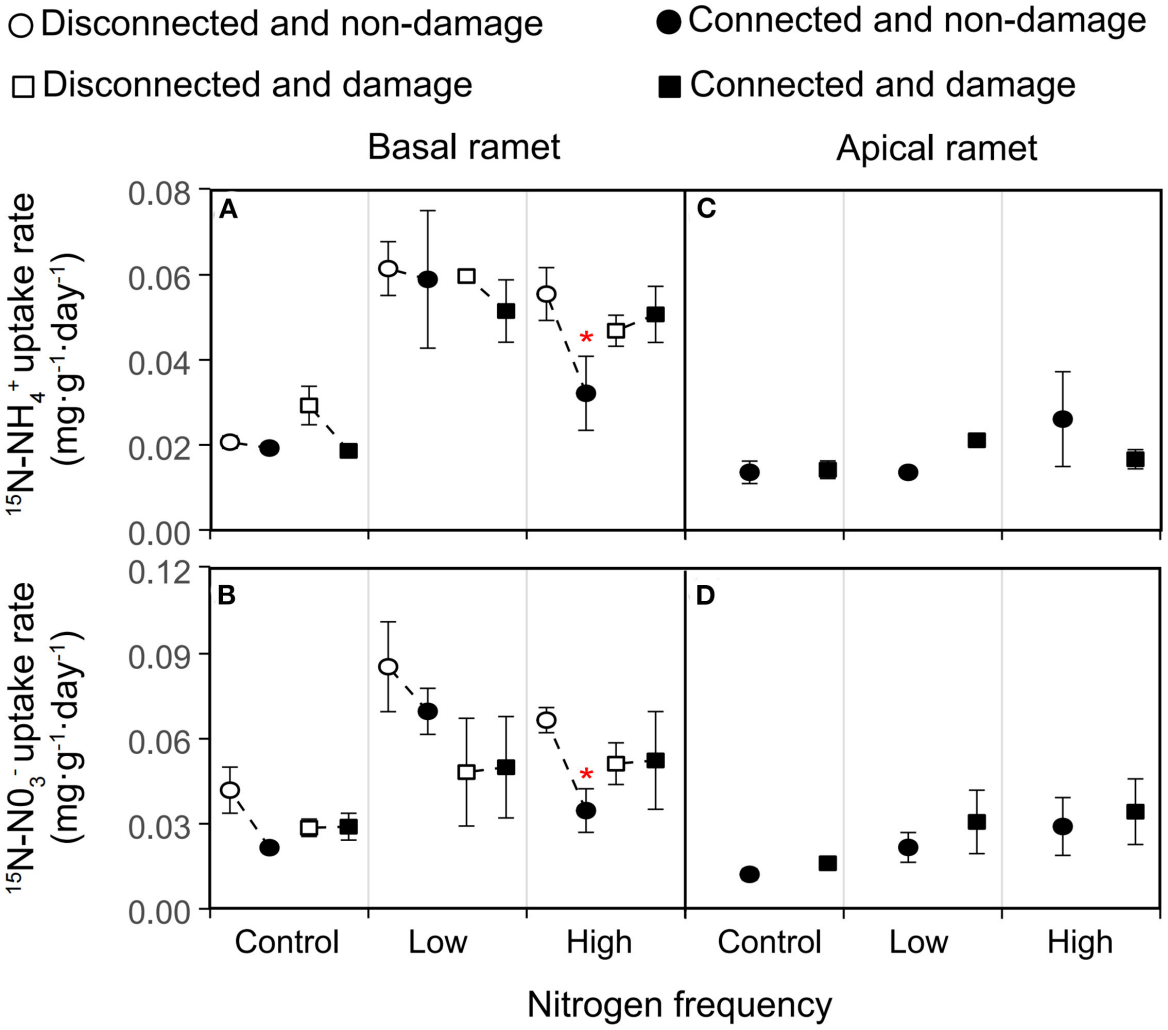
Effects of connection among ramets, N supply frequency, and damage on the ^15^N-${\text{NH}}_{4}^{+}$ uptake rate and ^15^N-${\text{NO}}_{3}^{-}$ uptake rate of the basal **(A,B)** and apical **(C,D)** ramets of *H. vulgaris*. Considering that there is no resource transfer when there is no connection between ramets, the isotope of apical ramets was not measured when there is no connection between ramets. Bars show the mean ± SE. The *p*-values with significant difference between disconnected and connected treatments under each N frequency and damage combination (linear contrast based on ANOVA) were marked above the connected treatment. *P = 0.01–0.05. Refer to Table 4 for ANOVAs.

A three-way ANOVA was used to test the effects of N frequency, connection, and damage (all factors were treated as fixed and categorical) on each measure of indexes of the entire clonal fragment and basal and apical ramets of *H. vulgaris* (Tables 1–**4**). Linear contrasts based on ANOVA were used to compare whether the effect of connection on various indexes of *H. vulgaris* was significant under each N frequency and damage treatment combination (Wang et al., 2020) (Figures 2–5). A one-way ANOVA was used to test the differences in the uptake rates of ^15^N-${\text{NH}}_{4}^{+}$ and ^15^N-${\text{NO}}_{3}^{-}$ of *H. vulgaris* (Supplementary Figure 1). The leaf mass and area of the entire clonal fragment, stem length, number of leaves, and total C and total N of the basal ramets, as well as the number of leaves, leaf area, total N, and the ^15^N uptake rates of the apical ramets were transformed to the natural logarithm or the square root before analysis as needed to improve homoscedasticity. Figures show untransformed data. All analyses were conducted using SPSS 22.0 (SPSS, Chicago, Illinois, USA).

**Table 1:** Effects of connected treatment, nitrogen (N) frequency, damage, and their interaction on the biomass, root mass, stem mass, and leaf mass of the entire clonal fragment **(A)**, basal ramets **(B)**, and apical ramets **(C)** of *Hydrocotyle vulgaris*.

	**Connection** **(C)**	**Nitrogen frequency** **(NF)**	**Damage** **(D)**	**C × NF**	**C × D**	**NF × D**	**C × NF × D**
	***F_**1,60**_***	***F_**2,60**_***	***F_**1,60**_***	***F_**2,60**_***	***F_**1,60**_***	***F_**2,60**_***	***F_**2,60**_***
**(A) Entire clonal fragment**							
Biomass	8.99^**^	10.49^***^	1.04^ns^	0.04^ns^	1.83^ns^	0.80^ns^	0.29^ns^
Root mass	9.45^**^	10.62^***^	0.89^ns^	0.01^ns^	0.36^ns^	2.70^ns^	1.27^ns^
Stem mass	8.38^**^	6.40^**^	0.97^ns^	0.03^ns^	1.65^ns^	0.76^ns^	0.31^ns^
Leaf mass	9.39^**^	29.00^***^	1.18^ns^	0.35^ns^	3.00^ns^	0.44^ns^	0.11^ns^
**(B) Basal ramets**							
Biomass	4.52^*^	15.62^***^	1.49^ns^	0.91^ns^	0.30^ns^	1.50^ns^	0.56^ns^
Root mass	3.95^ns^	12.80^***^	1.29^ns^	0.41^ns^	0.23^ns^	2.30^ns^	2.60^ns^
Stem mass	5.25^*^	9.97^***^	1.10^ns^	0.91^ns^	0.38^ns^	1.21^ns^	0.47^ns^
Leaf mass	2.22^ns^	36.87^***^	2.66^ns^	0.94^ns^	0.48^ns^	1.85^ns^	0.59^ns^
**(C) Apical ramets**							
Biomass	5.89^*^	0.68^ns^	0.05^ns^	1.96^ns^	2.54^ns^	0.36^ns^	0.61^ns^
Root mass	7.46^**^	0.84^ns^	0.01^ns^	0.99^ns^	2.94^ns^	0.99^ns^	0.78^ns^
Stem mass	4.77^*^	0.64^ns^	0.16^ns^	1.46^ns^	2.17^ns^	0.50^ns^	0.46^ns^
Leaf mass^a^	11.28^**^	1.15^ns^	0.00^ns^	2.64^ns^	3.05^ns^	0.08^ns^	0.94^ns^

*Numbers are ANOVA F-values. ns, P > 0.05*;

* *P = 0.01–0.05*;

** *P = 0.001–0.01; and*

*** *P < 0.001*.

a *indicaes that the data have undergone square transformation*.

## Results

### Morphological Traits of the Entire Clonal Fragment of *H. vulgaris*

The connection between ramets and a higher N frequency had significantly increased the biomass, root mass, stem mass, leaf mass, stem length, number of nodes, number of leaves, and leaf area of the entire clonal fragment (*P* < 0.05; Figures 2A–D, 3A–D; Tables 1A, 2A). However, damage insignificantly decreased all growth traits of the entire clonal fragment (*P* > 0.05; Figures 2A–D, 3A–D; Tables 1A, 2A). Besides, the connection and N frequency interactive effect significantly increased the leaf area of the entire clonal fragment (*P* < 0.05; Figure 3D; Table 2A).

**Table 2:** Effects of connected treatment, N frequency, damage, and their interaction on the stem length, number of nodes, number of leaves, and leaf area of the entire clonal fragment **(A)**, basal ramets **(B)**, and apical ramets **(C)** of *H. vulgaris*.

	**Connection (C)**	**Nitrogen frequency** **(NF)**	**Damage (D)**	**C × NF**	**C × D**	**NF × D**	**C × NF × D**
	***F_**1,60**_***	***F_**2,60**_***	***F_**1,60**_***	***F_**2,60**_***	***F_**1,60**_***	***F_**2,60**_***	***F_**2,60**_***
**(A) Entire clonal fragment**							
Stem length	10.18^**^	11.30^***^	0.06^ns^	0.18^ns^	1.44^ns^	0.52^ns^	0.13^ns^
Number of ramets	8.34^**^	6.40^**^	0.05^ns^	0.16^ns^	0.34^ns^	1.13^ns^	0.69^ns^
Number of leaves	4.61^*^	17.75^***^	0.25^ns^	0.45^ns^	1.04^ns^	0.70^ns^	0.63^ns^
Leaf area^b^	16.10^***^	53.91^***^	0.69^ns^	3.38^*^	2.23^ns^	0.29^ns^	0.12^ns^
**(B) Basal ramets**							
Stem length ^b^	6.71^*^	21.77^***^	1.63^ns^	1.69^ns^	1.01^ns^	0.32^ns^	1.80^ns^
Number of nodes	4.33^*^	11.39^***^	0.11^ns^	0.42^ns^	0.08^ns^	0.99^ns^	1.47^ns^
Number of leaves ^a^	0.97^ns^	34.79^***^	0.71^ns^	0.96^ns^	0.10^ns^	0.97^ns^	2.34^ns^
Leaf area	0.52^ns^	39.95^***^	1.20^ns^	0.93^ns^	0.06^ns^	2.28^ns^	0.52^ns^
**(C) Apical ramets**							
Stem length	5.52^*^	0.51^ns^	0.15^ns^	2.04^ns^	3.00^ns^	0.33^ns^	0.61^ns^
Number of nodes	3.85^ns^	0.26^ns^	0.56^ns^	1.96^ns^	1.53^ns^	0.42^ns^	0.41^ns^
Number of leaves ^a^	5.94^*^	0.14^ns^	3.10^ns^	2.17^ns^	3.19^ns^	0.14^ns^	0.86^ns^
Leaf area ^b^	27.85^***^	2.54^*^	0.05^ns^	0.22^ns^	3.19^ns^	0.05^ns^	1.57^ns^

*Numbers are ANOVA F-values. ns, P > 0.05*;

* *P = 0.01–0.05*;

** *P = 0.001–0.01; and*

*** *P < 0.001*.

a *indicaes that the data have undergone square transformation, and*

b *indicaes that the data have undergone logarithmic transformation*.

A linear contrast analysis revealed that, under the treatment of high N frequency at the basal ramet and apical ramet damage, the connection between ramets significantly increased the total biomass, leaf mass, and leaf area of the entire clonal fragment (*P* < 0.05; Figures 2A,D, 3D). Moreover, under the treatment of no N supply at the basal ramet and apical ramet damage, the connection between ramets significantly increased the root mass, stem mass, and leaf area of the entire clonal fragment (*P* < 0.05; Figures 2B,C, 3D).

In addition, the basal and apical ramets of *H. vulgaris* had different responses to each treatment (Figures 2, 3; Tables 1, 2).

### Morphological Traits of the Basal Ramets of *H. vulgaris*

The connection between ramets significantly increased the total biomass, stem mass, stem length, and number of nodes of the basal ramets (*P* < 0.05; Figures 2E,G, 3E,F; Tables 1B, 2B). At the same time, a higher N frequency significantly increased the biomass, root mass, stem mass, leaf mass, stem length, number of nodes, number of leaves, and leaf area of the basal ramets (*P* < 0.05; Figures 2E–H, 3E–H; Tables 1B, 2B). However, damage to the basal ramets had no significant effect on all growth traits of the basal ramets (*P* > 0.05; Figures 2E–H, 3E–H; Tables 1B, 2B).

A linear contrast analysis revealed that, under the treatment of no N supply at the basal ramet and apical ramet damage, the connection between ramets significantly increased the total biomass, root mass, stem mass, leaf mass, stem length, and number of nodes in the basal ramets (Figures 2E–H, 3E,F).

### Morphological Traits of the Apical Ramets of *H. vulgaris*

The connection between ramets significantly increased the total biomass, root mass, stem mass, leaf mass, stem length, number of leaves, and leaf area of the *H. vulgaris* apical ramets (*P* < 0.05; Figures 2I–L, 3I,K,L; Tables 1C, 2C). A higher N frequency significantly increased the leaf area in apical ramets (*P* < 0.05; Figure 3L; Table 2C). However, damage to the apical ramets had no significant effect on all growth traits (*P* > 0.05; Figures 2I–L, 3I–L; Tables 1C, 2C).

Based on the linear contrast analysis, under the treatment of high N frequency at the basal ramet and apical ramet damage, connected ramets significantly increased all the growth traits of the *H. vulgaris* apical ramets (*P* < 0.05; Figures 2I–L, 3I–L).

### Physiological Traits of the Basal Ramets of *H. vulgaris*

The growth and physiological indexes of *H. vulgaris* had a similar trend in all the treatments (Figures 2–**5**). The connection between ramets significantly increased the total C of basal ramets (*P* < 0.05; Figure 4A; Table 3A), but there was no significant effect on the total N (*P* > 0.05; Figure 4B; Table 3A). However, the high N frequency significantly increased the total C and N of the basal ramets (*P* < 0.05; Figures 4A,B; Table 3A). At the same time, damage to the ramets had no significant effect on the total C and N of *H. vulgaris* basal ramets (*P* > 0.05; Figures 4A,B; Table 3A). In addition, the interactive effect between connection and N frequency and damage, connection, and N frequency significantly affected the total C of the *H. vulgaris* basal ramets (*P* < 0.05; Figure 4A; Table 3A).

**Table 3:** Effects of connected treatment, N frequency, and damage and their interaction on the total carbon and total N of the basal **(A)** and apical **(B)** ramets of *H. vulgaris*.

	**Connection (C)**	**Nitrogen frequency** **(NF)**	**Damage** **(D)**	**C × NF**	**C × D**	**NF × D**	**C × NF × D**
	***F_**1,60**_***	***F_**2,60**_***	***F_**1,60**_***	***F_**2,60**_***	***F_**1,60**_***	***F_**2,60**_***	***F_**2,60**_***
**(A) Basal ramets**							
Total carbon^b^	6.13^*^	26.76^***^	3.78^ns^	2.54^ns^	2.10^ns^	0.615^ns^	1.24^ns^
Total nitrogen^a^	3.56^ns^	14.85^***^	1.33^ns^	4.15^*^	1.26^ns^	1.33^ns^	1.66^*^
**(B) Apical ramets**							
Total carbon	0.632^ns^	5.91^**^	0.03^ns^	2.05^ns^	2.53^ns^	0.30^ns^	0.52^ns^
Total nitrogen^a^	1.29^ns^	3.77^*^	0.13^ns^	0.34^ns^	3.63^ns^	0.47^ns^	0.67^ns^

*Numbers are ANOVA F-values. ns, P > 0.05*;

* *P = 0.01–0.05*;

** *P = 0.001–0.01; and*

*** *P < 0.001*.

a *indicaes that the data have undergone square transformation, and*

b *indicaes that the data have undergone logarithmic transformation*.

Based on the linear contrast analysis, under the treatment of no N supply at the basal ramet and apical ramet damage, connected ramets significantly increased total C and N of the basal ramets (*P* < 0.05; Figures 4A,B).

### Physiological Traits of the Apical Ramets of *H. vulgaris*

The connection between ramets slightly increased the total C of apical ramets (*P* > 0.05; Figures 4C,D; Table 3B). In addition, the N frequency significantly increased the total C (*P* < 0.05; Figure 4C; Table 3B) and decreased the total N of the *H. vulgaris* apical ramets (*P* < 0.05; Figure 4D; Table 3B). In contrast, damage to ramets had no significant effect on the total C and N of the apical ramets (*P* > 0.05; Figures 4C,D; Table 3B).

The linear contrast analysis revealed that, under the treatment of high N frequency at the basal ramets and apical ramet damage, connected ramets significantly increased the total C and N of the apical ramets (*P* < 0.05; Figures 4C,D).

### The ^15^N-${\text{NH}}_{4}^{+}$ and ^15^N-${\text{NO}}_{3}^{\text{–}}$ Uptake Rates of the Basal and Apical Ramets of *H. vulgaris*

The ^15^N-${\text{NH}}_{4}^{+}$ and ^15^N-${\text{NO}}_{3}^{-}$ uptake rates of *H. vulgaris* had no significant difference (Supplementary Figure 1), and the response trend to each treatment was also consistent (Figure 5; Table 4). The connection between ramets had no significant effect on the ^15^N-${\text{NH}}_{4}^{+}$ and ^15^N-${\text{NO}}_{3}^{-}$ uptake rates of the basal ramets (*P* > 0.05; Figures 5A,B; Table 4A). Besides, the high N frequency significantly increased the ^15^N-${\text{NH}}_{4}^{+}$ and ^15^N-${\text{NO}}_{3}^{-}$ uptake rates of the basal ramets, while the low N frequency had no significant effect (*P* < 0.05; Figures 5A,B; Table 4A). Similarly, the damage had no significant effect on the ^15^N-${\text{NH}}_{4}^{+}$ and ^15^N-${\text{NO}}_{3}^{-}$ uptake rates of the basal ramets (*P* > 0.05; Figures 5A,B; Table 4A). Moreover, the linear contrast analysis revealed that, under a high N frequency and no damage treatment, the connection between ramets significantly decreased the ^15^N-${\text{NH}}_{4}^{+}$ and ^15^N-${\text{NO}}_{3}^{-}$ uptake rates of the basal ramets (*P* < 0.05; Figures 5A,B). In addition, N frequency and damage had no significant effect on ^15^N-${\text{NH}}_{4}^{+}$ and ^15^N-${\text{NO}}_{3}^{-}$ uptake rates of the apical ramets (*P* > 0.05; Figures 5C,D; Table 4B).

**Table 4:** Effects of connected treatment, N frequency, and damage and their interaction on the ^15^N-${\text{NH}}_{4}^{+}$ and ^15^N-${\text{NO}}_{3}^{-}$ uptake rates of the apical **(A)** and basal **(B)** ramets of *H. vulgaris*.

	**Connection** **(C)**	**Nitrogen frequency** **(NF)**	**Damage** **(D)**	**C × NF**	**C × D**	**NF × D**	**C × NF × D**
	***F_**1,60**_***	***F_**2,60**_***	***F_**1,60**_***	***F_**2,60**_***	***F_**1,60**_***	***F_**2,60**_***	***F_**2,60**_***
**(A) Basal ramets**							
^15^N-${\text{NH}}_{4}^{+}$ uptake rate	3.24^ns^	29.26^***^	0.14^ns^	0.12^ns^	0.27^ns^	0.60^ns^	2.20^ns^
^15^N-${\text{NO}}_{3}^{-}$ uptake rate	2.75^ns^	8.82^**^	2.40^ns^	0.14^ns^	3.33^ns^	2.03^ns^	0.13^ns^
**(B) Apical ramets**							
^15^N-${\text{NH}}_{4}^{+}$ uptake rate		1.57^ns^	3.14^ns^			1.84^ns^	
^15^N-${\text{NO}}_{3}^{-}$ uptake rate		1.84^ns^	0.62^ns^			0.13^ns^	

*Numbers are ANOVA F-values. ns, P > 0.05*;

** *P = 0.001–0.01; and*

*** *P < 0.001*.

## Discussion

### Effects of the Clonal Integration on *H. vulgaris* Traits

The connection among ramets significantly increased the biomass, stem mass, and total C of the basal ramets as well as the biomass, root mass, stem mass, leaf mass, stem length, leaf number, leaf area, and total C of the apical ramets (Figures 2–4). This implies that clonal integration significantly promoted the growth of *H. vulgaris* basal and apical ramet, with greater growth by the apical ramets. With clonal integration, the basal and apical ramets in clonal plants reallocate resources and reasonable division of labor that promote the growth of apical and basal ramets (Hartnett and Bazzaz, 1983; Roiloa and Retuerto, 2007; Zhang et al., 2009). Moreover, with clonal integration, ramets located in a high-resource habitat act as donor ramets, transferring some resources to those in low-resource habitats (Song et al., 2013; Wang et al., 2020). Therefore, clonal integration promoted the growth of the entire clonal fragment and basal and apical ramets of *H. vulgaris*, especially promoting the apical ramets more significantly.

### Effects of the N Supply With Different Frequencies on *H. vulgaris* Traits

Our results showed that the increase of N frequency promoted the growth of the entire clonal fragment of *H. vulgaris* (Figures 2, 3). N is an important nutrient to maintain plant growth (Gutiérrez, 2012; Song et al., 2012). Moreover, under small amounts of multiple applications of N, plants can absorb and use more N so as to grow better (Chang et al., 2014; Wu et al., 2019). Therefore, a higher N frequency can better promote the growth of *H. vulgaris*. In addition, the increase of N frequency significantly promoted the growth of basal ramets but had no significant effect on the apical ramets (Figures 2–4). This may be because the increase of N frequency increases the resources of the basal ramet environment. Since the resources captured by clonal ramets can be transferred between ramets (Roiloa and Retuerto, 2007; Zhang et al., 2009), allocating more resources to high resource ramets can make more full use of resources and improve the performance of the whole clonal plants (Ikegami et al., 2008; Huang et al., 2018). For example, under heterogeneous nutrient conditions, clonal integration generally increased biomass allocation to roots in the high resource ramets but decreased it in the low recourse ramets (Wang et al., 2021). Thus, the increase of N frequency only enhanced the growth of the basal ramets and had no significant effect on the apical ramets.

### Effects of the Ramet Damage on *H. vulgaris* Traits

The induction of damage on ramets had no significant effect on the *H. vulgaris* growth (Figures 2–4). Most plants have the ability to resist damage (Nguyen et al., 2016; Hakim et al., 2018; Lu et al., 2020; Qi et al., 2020). Besides, a meta-analysis of 32 invasive species found that invasive plants had stronger tolerance and compensation for damage (Zhang et al., 2018). In addition, more than one study shows that clonal plants can counter the local adverse conditions through reasonable resource allocation and division of labor among ramets, which also supports our results (Schmid et al., 1988; Alpert, 1999; You et al., 2014; Lyu et al., 2016; Liu et al., 2017a; Wang et al., 2017). Therefore, *H. vulgaris*, as an invasive clonal plant, has the ability to resist a certain degree of damage.

### Effects of the N Supply With Different Frequencies and Ramet Damage on *H. vulgaris* Clonal Integration

Under the treatment of high N frequency at the basal ramet and apical ramet damage, the connection between ramets more significantly improved the growth of the apical and entire clonal fragment (Figures 2–4). Most studies suggest that when resources are heterogeneous, clonal integration is beneficial to ramets in low-resource habitats (Friedman and Alpert, 1991; Song et al., 2013; Wang et al., 2017; Lin et al., 2018). Through clonal integration, the increase of available resources in the high resource ramets increases the resource availability in the low resource ramets and also improves the benefits of clonal integration to low resource ramets (Lin et al., 2018). Besides, in clonal plants, damage also stimulates the allocation of resources to damaged ramets to maintain their growth (Schmid et al., 1988; You et al., 2014; Lyu et al., 2016; Wang et al., 2020). Thus, under the treatment of high N frequency at the basal ramet and apical ramet damage, clonal integration significantly promoted the apical ramet growth. Moreover, the surplus resources of basal ramets are more fully utilized, and the ability of apical ramets to resist damage is also improved (Gao et al., 2014; Liu et al., 2017a). Therefore, clonal integration also provides more benefits to the entire clonal fragment.

It is worth noting that the low-frequency N supply and clonal integration had no significant impact on the apical ramet growth (Figures 2–4). This may be because, although low N frequency provides resources, it is limited. In previous studies, heterogeneous environments were often designed with high contrast, and the resources in high resource environments are often surplus, so the effect on the ramets located in low resource environments is significant (Guo et al., 2011; Wang et al., 2017). However, when resources are limited, clonal plants allocate the limited resources to ramets in the higher resource environment to capture more resources (Ikegami et al., 2008). Therefore, low-frequency N supply has no significant effect on the growth of apical ramets.

In addition, we found no N supply to the basal ramets and damage on the apical ramets, and the connection between ramets significantly increased the basal ramet growth, increasing the benefits of clonal integration on the basal ramets (Figures 2–4). This may be because the damage to the apical ramets serves as a signal that stimulates resource utilization at the basal ramets and makes an earlier stress response (Lyu et al., 2016; Wang et al., 2017). Another explanation is that clonal plants can enhance the ability to compensate for damage by concentrating ramets in less stressed patches in heterogeneous environments (Wise and Abrahamson, 2007; Sun et al., 2010).

Moreover, as we discussed earlier, when the basal ramet resources are limited, the resources will not be allocated to the apical ramets (Ikegami et al., 2008). However, when the resource availability of the basal ramets is high, some resources will be allocated to the apical ramets, which will reduce the benefits of clonal integration to the basal ramets (Wang et al., 2009; Song et al., 2013; Chen et al., 2015). Therefore, the effect of clonal integration on the basal ramets was significant only when there was no N addition at the base and the apical was damaged. In addition, a recent study showed that apical ramet damage inhibits the growth of basal ramets through clonal integration and reduces overall growth (Gao et al., 2021). Additionally, the treatment in this study is caused by parasitism of *Cuscuta australis* with a length of 15 cm (Gao et al., 2021). It may require more nutrients for growth, which caused greater damage to the clonal plants, exceeding the clonal ramets' resistance to damage, subsequently offsetting the clonal integration benefits.

### Effects of the Clonal Integration, N Supply Frequency, and Ramet Damage on the ^15^N-${\text{NH}}_{4}^{+}$ and ^15^N-${\text{NO}}_{3}^{\text{–}}$ Uptake Rates of *H. vulgaris*

Compared with no N supply, N supply increases the ^15^N-${\text{NH}}_{4}^{+}$ and ^15^N-${\text{NO}}_{3}^{-}$ uptake rates. N deposition increases the N uptake rate of clonal plant *Leymus chinensis*, and *L. chinensis* shows better advantages than *Stipa grandis* and *Cleistogenes squarrosa* in N deposition, which are similar to the study results by Cao et al. (2021). This suggests that the positive response in N absorption rate following N deposition may be an important factor supporting the diffusion and invasion of *H. vulgaris* (Liu et al., 2017b; Valliere et al., 2017; Wang and Chen, 2019). Besides, this study shows no significant difference in N absorption rate between the low- and high-frequency N supply, implying that the positive response in N absorption rate following N deposition was limited. This explains why frequent applications of small amounts of N significantly improved *H. vulgaris* growth.

In addition, under the conditions of no damage and high-frequency N supply, clonal integration significantly reduced the ^15^N-${\text{NH}}_{4}^{+}$ and ^15^N-${\text{NO}}_{3}^{-}$ uptake rates of the basal ramets and increased the uptake rates of the apical ramets. This is because ^15^N-${\text{NH}}_{4}^{+}$ and ^15^N-${\text{NO}}_{3}^{-}$ were applied 24 h before harvest, and the basal ramets accumulated a large amount of N in treatments with a high-frequency N supply and no damage. Some studies have shown that ramets growing in high resource patches usually transfer resources to low resource ramets to maintain the growth of low resource ramets (Guo et al., 2011; Wang et al., 2017).

A few potential limitations should be considered within the context of this study. First, the experiment is a pot control experiment. The response of *H. vulgaris* to N deposition and ramet damage in the field may be more complex than our research. Second, N was added to the basal ramets, and the damage was in the apical ramets in the experiment. In the field, the heterogeneity of N content and ramet damage is usually random. Whether basal ramet damage and N addition to apical ramets will significantly affect the results is uncertain. Finally, this research mainly focuses on the response of *H. vulgaris* to N deposition, and only one degree of ramet damage treatment is designed. How *H. vulgaris* responds to more severe damage is unclear. Future studies are encouraged to design more damage levels. In addition, the basal and apical ramets should be treated with N addition and damage, respectively, to gain a comprehensive understanding of the response of *H. vulgaris* to N deposition and ramet damage.

## Conclusion

Both clonal integration and higher frequency N supply promoted the growth of the entire clonal fragment of *H. vulgaris*, and clonal integration more significantly promoted the growth of apical ramets. However, higher frequency N supply more significantly promoted the growth of basal ramets. Ramet damage had no significant effect on the growth of *H. vulgaris*. Besides, the heterogeneous N supply with high frequency and ramet damage increased the clonal integration benefits in ramets in a given environment, subsequently benefiting the entire *H. vulgaris* clonal plant. Moreover, the size of differences in heterogeneous resources affected the resource allocation among ramets. In addition, the uptake rates of ${\text{NH}}_{4}^{+}$ and ${\text{NO}}_{3}^{-}$ of *H. vulgaris* had no significant difference, and N supply increased the uptake rates of ${\text{NH}}_{4}^{+}$ and ${\text{NO}}_{3}^{-}$ of the basal ramets. Taken together, our study increases the understanding of the growth of invasive clonal plants and their clonal integration in response to N deposition and ramet damage.

## Data Availability Statement

The raw data supporting the conclusions of this article will be made available by the authors, without undue reservation.

## Author Contributions

H-LL and Y-NM: conception and design of this study. KS: drafting the manuscript and analysis and interpretation of data. J-FC and YZ: revising the manuscript critically for important intellectual content. S-HA, Y-LS, and L-JY: acquisition of data. KS, J-FC, YZ, Y-NM, S-HA, Y-LS, L-JY, and H-LL: approval of the version of the manuscript to be published. All authors contributed to the article and approved the submitted version.

## Funding

This study was funded by the National Key Research and Development Program of China (Grant No. 2021YFC2600400), the China Major Science and Technology Program for Water Pollution Control and Treatment (2017ZX07602-004-003), the National Key Research and Development Program of China (2017YFC0505905), the Fundamental Research Funds for the Central Universities (2015ZCQ-BH-01), the National Natural Science Foundation of China (31470475), and the Beijing Municipal Education Commission's financial support through Innovative Transdisciplinary Program Ecological Restoration Engineering.

## Conflict of Interest

The authors declare that the research was conducted in the absence of any commercial or financial relationships that could be construed as a potential conflict of interest.

## Publisher's Note

All claims expressed in this article are solely those of the authors and do not necessarily represent those of their affiliated organizations, or those of the publisher, the editors and the reviewers. Any product that may be evaluated in this article, or claim that may be made by its manufacturer, is not guaranteed or endorsed by the publisher.
