# Supplementary material for: Heterogeneous Nitrogen Supply With High Frequency and Ramet Damage Increases the Benefits of Clonal Integration in Invasive Hydrocotyle vulgaris
Source: Front Plant Sci. 2022 Apr 29;13:825492. doi: 10.3389/fpls.2022.825492 (PMC9100825; doi:10.3389/fpls.2022.825492)
Supplement: Supplementary file 1 [file Data_Sheet_1.docx]

Supplementary Material

## Supplementary Figures

**
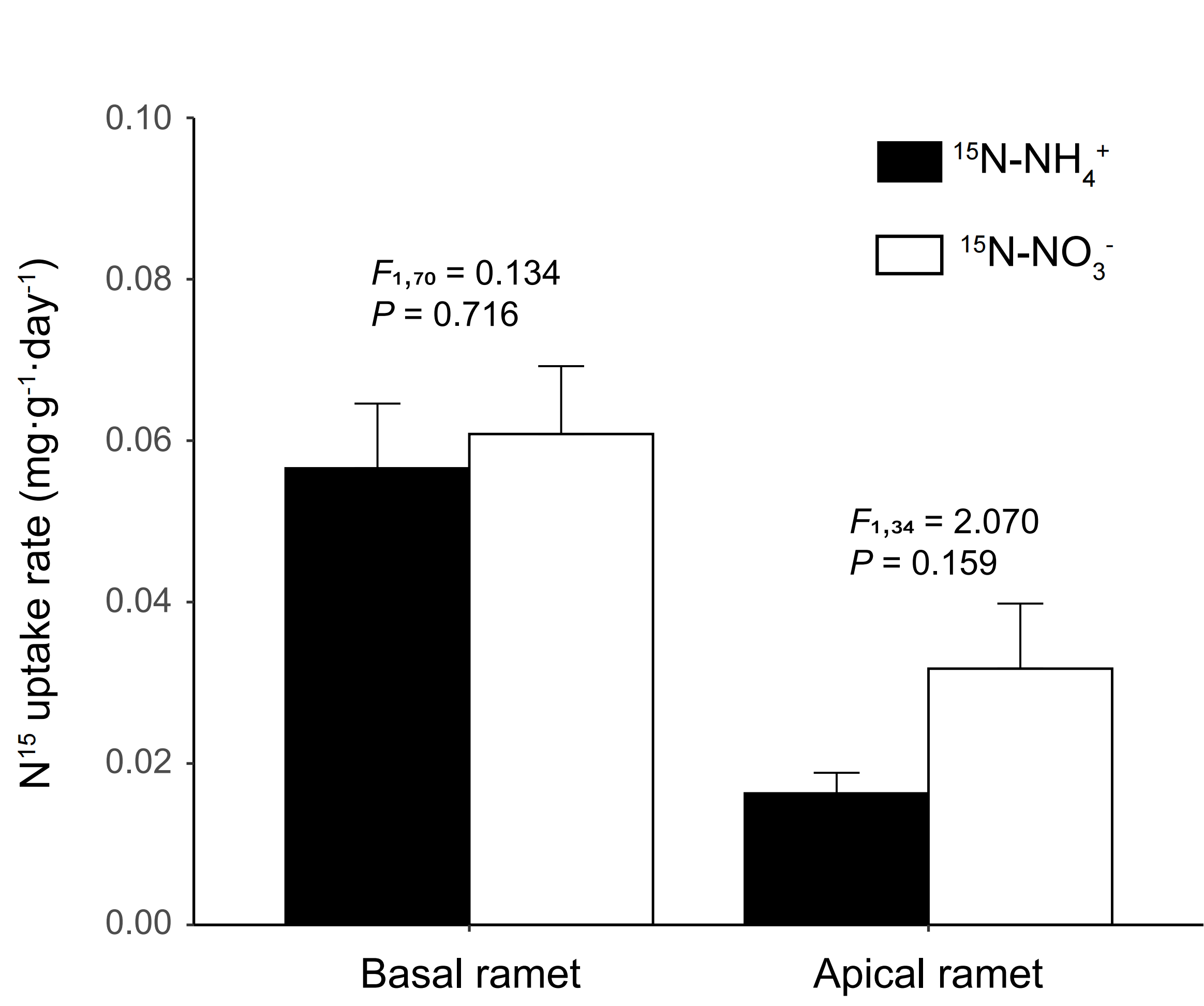
**

**Supplementary Figure 1.** The ^15^N-NH_4_^+^ and ^15^N-NO_3_^-^ uptake rates of the basal and apical ramets of *Hydrocotyle vulgaris.* Bars represent the mean ± SE. The results of ANOVA were marked above the bars.
